# From Discovery to Production: Biotechnology of Marine Fungi for the Production of New Antibiotics

**DOI:** 10.3390/md14070137

**Published:** 2016-07-21

**Authors:** Johanna Silber, Annemarie Kramer, Antje Labes, Deniz Tasdemir

**Affiliations:** 1 GEOMAR Helmholtz Centre for Ocean Research Kiel, Marine Natural Products Chemistry Research Unit, GEOMAR Centre for Marine Biotechnology (GEOMAR-Biotech), Am Kiel-Kanal 44, Kiel 24106, Germany; jsilber@geomar.de (J.S.); akramer@geomar.de (A.K.), alabes@geomar.de (A.L.); 2 Faculty of Mathematics and Natural Sciences, University of Kiel, Christian-Albrechts-Platz 4, Kiel 24118, Germany

**Keywords:** marine biotechnology, transfer to stirred tank reactor, full fermentative process, semi-synthesis, biological derivatisation, filamentous fungi, bioprocess development, heterologous expression, genetic and metabolic engineering, downstream processing

## Abstract

Filamentous fungi are well known for their capability of producing antibiotic natural products. Recent studies have demonstrated the potential of antimicrobials with vast chemodiversity from marine fungi. Development of such natural products into lead compounds requires sustainable supply. Marine biotechnology can significantly contribute to the production of new antibiotics at various levels of the process chain including discovery, production, downstream processing, and lead development. However, the number of biotechnological processes described for large-scale production from marine fungi is far from the sum of the newly-discovered natural antibiotics. Methods and technologies applied in marine fungal biotechnology largely derive from analogous terrestrial processes and rarely reflect the specific demands of the marine fungi. The current developments in metabolic engineering and marine microbiology are not yet transferred into processes, but offer numerous options for improvement of production processes and establishment of new process chains. This review summarises the current state in biotechnological production of marine fungal antibiotics and points out the enormous potential of biotechnology in all stages of the discovery-to-development pipeline. At the same time, the literature survey reveals that more biotechnology transfer and method developments are needed for a sustainable and innovative production of marine fungal antibiotics.

## 1. Need for New Antibiotics

The discovery of the first antibiotic penicillin from the mold *Penicillium notatum* by Sir Alexander Fleming opened up a completely new era of chemotherapy, thereby changing the quality of human life. Ever since the development of penicillin in 1940s that initiated the golden era of further natural antibiotics from *Streptomyces* species, we have benefited from antibiotics from diverse chemical classes against pathogenic bacteria [1]. More than 350 agents so far have reached the world market as antimicrobials [2]. They include natural products, semi-synthetic antibiotics and synthetic chemicals [2,3]. The wide use of antibiotics, however, has resulted in the development of resistant microbes due to the evolutionary selection pressure driven by antibiotics [4]. The number of effective therapeutic measures against life-threatening bacterial and fungal infections has fallen dramatically because of the emerging multidrug-resistant (MDR) pathogens. Nowadays, infectious diseases are listed as the second leading cause of death worldwide and are regarded as a very major global societal challenge of the century [5,6]. Alarmingly, many pharmaceutical companies have significantly reduced or completely terminated R&D programmes on antibiotics. Drug discovery pipelines are almost empty, with only a few new drug candidates in registration or in development [5]. Only recently, the dramatic increase of incidence of bacterial MDR infections has led to a revival of antibiotic programs. Further measures and strategies are urgently needed worldwide to tackle the drug resistance and promote research in antibiotic drug discovery, including those from natural sources [7].

Microbial sources have been very prolific producers of thousands of natural antibiotics. More than half of all antibiotics are produced by actinomycetes, 10%–15% by non-filamentous bacteria and about 20% by filamentous fungi [8]. Still, this possibly only represents a small portion of the repertoire of microbial bioactive compounds, as drug discovery efforts often involve a specific focus, either on taxonomic groups or on habitats. In that sense, marine ecosystems have remained almost untapped although marine microorganisms can accumulate structurally unique bioactive natural products that are not found in terrestrial counterparts. A detailed analysis by Kong et al. showed that large portions of marine scaffolds are novel [9]. Auranomides A and B, quinazolin-4-ones substituted with a pyrrolidin-2-iminium moiety from the marine-derived fungus *Penicillium aurantiogriseum,* or aspergilols A and B with a C–C fusion of an anthraquinone and orcinol unit from a deep-sea *Aspergillus versicolor* represent new scaffolds from marine habitats [10,11]. Secondary metabolites that are exclusively produced by marine organisms are thought to facilitate their adaptation and survival in the marine environments characterised by very special conditions [12]. The microbial biodiversity of marine environments is enormous, but has not yet been explored and characterised—neither taxonomically nor chemically. Latest estimates calculate that probably less than 0.1% of the marine microbial diversity has been studied [13]. To our knowledge, no specific estimates are available for marine fungi.

Since the 1980s, the number of antibiotic compounds isolated from marine fungi is rapidly increasing. Most sampled sources are marine algae, sponges, and mangroves that have been shown to harbour highly talented associated fungi in terms of natural product production. The ecological reasons for this talent remain hypothetical for most cases [14,15]. Although marine fungi currently attract a good deal of research efforts, most of the studies are limited to the description of new chemical structures with in vitro biological activities. The discovery of new compounds is only the very first step of the long, expensive, and risky drug discovery pipeline, which requires large supply of the metabolite of interest. Converting the discovery of a lead compound to production at sufficient amounts for development, or processing into clinical trials is unfortunately far from being advanced. Biotechnology can offer realistic routes to the adequate supply and promotion of new compounds into marketable drugs. In this review, we report on the biotechnological approaches using marine fungi for the production of new antibiotics.

## 2. Marine Fungi as a Promising Source to Meet the Need for New Antibiotics

The discovery of penicillin fuelled the exploration and exploitation of microorganisms as excellent sources for antibiotics [1]. This led to a boost in development of biotechnological techniques, which were subsequently transferred to processes for a large variety of products [16]. The past decade witnessed a renewed interest in fungi for development of anti-infective lead compounds, enhancing the awareness of the importance of biotechnology. Unfortunately, the extremely low cultivability (1%) of microorganisms in standard laboratory conditions [17] restricts the discovery and subsequent biotechnological development of further antibacterial compounds possibly encoded in so-far-unknown fungi. The culture-independent molecular description of microbes from a number of natural habitats has uncovered a hitherto unknown microbial wealth, showing a new dimension of fungal diversity by revealing the presence of novel environmental marine phylotypes. To name only a few: deep sea *Pezizomycotina*, as an environmental clone group (PCG), and novel environmental sequences from marine anoxic vent habitats grouped together into the hydrothermal and/or anaerobic fungal group (Hy-An Group) within Basidiomycota and Ustilaginomycotina, for review see [18]. The absence of fungal isolates of these groups and limited availability of marine fungal strains in culture collections have caused insufficient exploration of biological diversity so far. In order to increase the chemodiversity, the biodiversity has to be increased accordingly. In addition, many biosynthetic gene clusters remain dormant or under-expressed in artificial laboratory culture conditions [19]. This leads to another restriction, i.e., the frequent replication of already known compounds, which sets a severe limit [20]. It can be assumed that the opportunity for discovery of new bioactive molecules in marine fungi could exceed any organisms from other ecosystems, as the oceans represent highly competitive environment with a longer evolutionary history and an untapped biodiversity. Research in recent years has led to significant improvements in the existing techniques for discovery of new compounds and thereby the available chemical space. Efforts undertaken in that context include the stimulation of the so-called “silent” gene clusters by external stimuli such as modification of all culture parameters (One-Strain-Many-Compounds = OSMAC approach [21]), mimicking environmental conditions [22,23] or the co-culturing with other microbes [24]. Different types of stress (e.g., UV) and competitive environments can also be applied, for a review see [23].

In the 1950s, cephalosporin C, a β-lactam type natural antibiotic, was discovered from a *Cephalosporium* (nowadays *Acremonium*) species obtained off the Sardinian coast [25,26]. Cephalosporin C represents the first fungal antibiotic from a marine environment. In the late 1970s, gliotoxin was identified as a new type of the antibiotic diketopiperazine produced by an *Aspergillus* sp. strain isolated from marine mud of the Seto Inland Sea. It was the first antimicrobial compound of this type obtained from a fungus originating from deep-sea sediments [27]. Later, gliotoxin and related diketopiperazines were shown to be produced by many marine and terrestrial fungi [27]. The first antibiotic compound from a marine yeast was indanonaftol A, a spiro-indanone derivative from a marine *Aureobasidium* sp. with weak activity against Gram-positive bacteria [28,29]. A steadily increasing number of new, active fungal natural products have since been identified from the marine environment, proving them to be a prolific source for bioactive compounds including antibiotics. While a total number of 272 compounds had been described from marine fungi until 2002 [20], almost 200 new natural products were identified from this source in 2009 alone [14]. This trend seems to continue: Blunt et al. listed over 200 natural products from marine-sourced fungi (excluding those from mangroves) in the year 2013, 5% of which with antibacterial properties against different *Staphylococcus* and *Vibrio* species, *Escherichia* *coli* and *Xanthomonas* *campestris* [30,31]. Cuomo et al. observed higher hit rates in marine isolates as compared to terrestrial ones, while cultivating approximately 1500 terrestrial and the same order of marine strains for evaluation of their antimicrobial potential [32]. 

Fungal marine natural products include a large diversity of structural classes and a wide range of substituent patterns resulting in some remarkable antibacterial and other bioactivities. Ascochital, for instance, is a fairly potent aromatic aldehyde against *Bacillus subtilis* with a minimal inhibitory concentration (MIC) of 0.5 μg/mL. This compound was isolated from the marine ascomycete *Kirschsteiniothelia maritima* derived from a submerged wood sample [20]. The aminolipopeptides trichoderins A, A1, and B from a marine *Trichoderma* sp. (isolated from an unidentified marine sponge) exhibited notable activity against *Mycobacterium smegmatis*, *M. bovis* BCG and *M. tuberculosis* H37Rv. The trichoderins display activity against the active and dormant *Mycobacterium* test strains with MICs in the range of 0.02–2.0 μg/mL [33]. Most illustrative example for the potential of marine fungi as producers of promising antibiotic candidates is pestalone. This chlorinated benzophenone was obtained from the mixed cultivation of a marine *Pestalotia* sp. (isolated from the surface of the Bahamian brown alga *Rosenvingea* sp.) with an unidentified marine bacterium. Pestalone showed highly potent activity against methicillin-resistant *Staphylococcus aureus* and vancomycin-resistant *Enterococcus faecium* with MIC values of 37 ng/mL and 78 ng/mL, respectively [34].

## 3. Biotechnology for Sustainable Production of New Antibiotics

In contrast to macro-organisms, micro-organisms have the advantage of feasible and sustainable production of large quantities of anti-infective natural products by large-scale cultivation at reasonable costs [35]. This is reflected in the proportion of fermentation products of worldwide antibiotic manufacture with total amounts of 100,000 tons, including 60,000 tons of penicillins, 5,500 tons of tetracyclines and 2,500 tons of cephalosporins. Antibiotics that are natural or derived from natural products include β-lactam antibiotics such as ampicillin (5,000 tons per year), cephalexin (4,000 tons), amoxicillin (16,000 tons), and cefadroxil (1,000 tons). Macrolides at high tonnage comprise azithromycin (1,500 tons) and clarithromycin (1,500 tons). Glycopeptides, such as vancomycin and teicoplanin, are produced at a total of 9,000 tons [8]. Nevertheless, the success stories in marine biotechnology are far fewer than in all other fields of commercial biotechnology [36]. Out of the many hundreds of bioactive compounds reported from marine fungi, only few reached commercialisation. The lack of transfer from the discovery stage to a proper biotechnological process chain has limited—at least partly—the availability of sufficient quantities of the compounds either for clinical trials or for early-stage lead development such as modification by chemical or biocatalytic means. 

**Figure 1:**
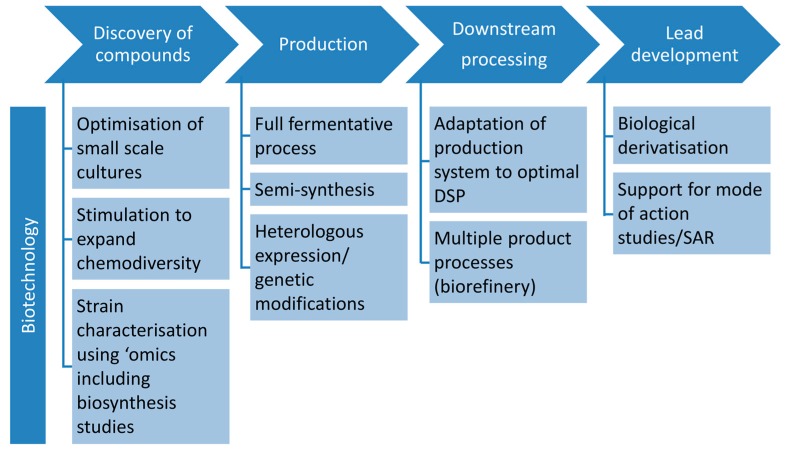
Biotechnological approaches at different steps of the process chain for developing antibiotics from marine fungi. Abbreviations: SAR, structure-activity relationship; DSP, downstream processing.

Similar to the biopharmaceutical biotechnology sector, bioprocess engineering in marine biotechnology follows the path from discovery to commercialisation with a variety of possible starting points and approaches, which can be used to take the compound to the next developmental step. A full value chain remains theoretical, as quantitative biotechnological engineering studies on marine fungi are virtually non-existing in the literature. As illustrated by Figure 1, biotechnology has a vast potential for sustainable production of antibiotics from marine fungi, starting from methods that help to expand and understand the chemical space in a targeted manner, via classical full fermentative and semi-synthetic processes to metabolic engineering manipulating the genetic background as a basis for generation of “biological” derivatives.

In the following, we describe the current state of the various possibilities and approaches along the marine biotechnological process chain in the field of antibiotics from marine fungal producers (Table 1, Figure 2). The review covers all available marine fungal antibiotic compounds for which at least one biotechnological approach has been published since March 2016. Table 1 gives an overview, which will be described in more detail in the following chapters. Strategies to expand the chemical space by improving chemical and microbiological methods were not considered as biotechnology approaches.

### 3.1. Role of Biotechnology in Discovery

For discovery of an antibacterial product, usually a specific species is selected, the product is extracted, screened for antibacterial activity, subjected to (bioactivity-guided) isolation process and finally the natural product is obtained in a highly pure form. This type of natural product discovery is slow, tedious, labour intensive and inefficient [59]. Recent advancements in the dereplication and other analytical methods have led to more efficient natural product discovery. Biotechnology can as well contribute to the rapid discovery of metabolites from marine fungi by a number of approaches [60], overcoming random approaches such as OSMAC:

- Controlled miniaturisation for increased screening throughput. Cultivation in small-scale fermentation (mL ranges) systems, e.g., microtiter plates, has been proven to be feasible for marine fungi [61,62,63] and used in screening for antibiotics of filamentous fungi (in deep well microtiter plates [64]). Controlled process development as the biotechnological approach is possible in specialised miniaturised fermentation systems such as “System Duetz” [65] or “BioLector” [66,67]. They allow enlarging the number of tested cultivation conditions, as well as to screen strains or mutant libraries in a targeted and efficient way. A mutant library established from a marine fungus has recently been screened for anticancer natural products [63]. This bioactivity-independent approach can be easily transferred to the antibiotic field. For detailed information on the application of microtiter plates as mini-bioreactors the reader is referred to the review by Duetz [68]. Such systems are necessary for the efficient application of statistical approaches for process optimisation (Design of Experiment, DoE, [69,70]).
- Targeted stimulation of strains to expand chemodiversity. A proper understanding of fungal producers and their ecological role can help to find the appropriate production conditions and extend the chemical diversity of their constituents [71]. Standard approaches in recent years focussed on triggering a strain by as many parameters (randomly chosen) as possible (OSMAC) [21]. More strategic approaches would include targeted mixed fermentations based on genetic and ecological knowledge as used in the food biotechnology to enhance enzyme production. The full potential of such approaches of ecologically or genetically-based biotechnology needs to be proven in the future.
- Strain characterisation using ‘omics’ techniques. Comprehensive knowledge from genome to metabolome level contributes to a general understanding of the fungal potential in drug discovery but also to concrete optimisation strategies for biotechnological processes. Especially the analyses on proteome level (proteomics) may deliver valuable insights into the producing cell, underlying regulatory processes and angles for metabolic engineering [72]. Transcriptomics, proteomics, and secretomics can be applied to elucidate the metabolic state of a cell on all levels of gene regulation and to indicate regulation sites on DNA, RNA, and protein level. Based on such knowledge, conditions required to induce expression of the full biosynthetic potential of an organism can be established and further be controlled [60]. Until now, no such example is available for production of antibiotics by marine fungi, but this approach is considered as one of the major directions for future research. Current examples from a related field, anticancer compounds, already show how powerful these tools may be: A comparative proteome study on a marine *Microascus brevicaulis* revealed how the biotechnological fermentation process should be controlled in order to increase the production of the anti-cancer compounds scopularides A and B [73]. Furthermore, fluxomics, which determine the metabolic flux of primary molecules during primary and secondary metabolism in a quantitative manner, is a powerful tool to display the conversation of nutrient source into products or by-products [74]. This knowledge can be used to design fermentation conditions or to engineer the underlying pathways by means of genetic modification. Metabolomics, finally, identifies the global metabolite profile in both qualitative and quantitative manner.

### 3.2. Role of Biotechnology in Production 

The production of natural products in sufficient amounts is crucial for drug development and can be achieved by a number of biotechnological approaches. Most publications referring to biotechnology of marine fungal antibiotics have contributed to that part of the production chain (Table 1). They cover (i) full fermentative processes using the natural producer; (ii) semi-synthetic approaches (i.e., fermentation to produce either a precursor molecule or modifying a synthetic product by means of bioconversion), and (iii) heterologous production in genetically-modified hosts. In the following sections, we describe these directions in detail.

#### 3.2.1. Production Using Full Fermentative Processes

Although fungi have a long history of use in food production, the implementation of the industrial-scale fermentation of penicillin reflects the breakthrough of filamentous fungal biotechnology. The well-accepted suitability of large-scale production of microorganisms in contrast to macro-organisms is based on the possibility to transfer respective microbial producer strains into an established biotechnological production process. Ng et al. state that “for the cultivable microorganisms, the problem of isolation of enough raw natural product materials is easily solved by adopting large-scale cultivation or fermentation when using different approaches to optimise the culture medium for the enhanced production of target biomolecules” [59]. However, research on the optimisation of marine fungal fermentation in bioreactors is insufficient [36]. Most production strategies were established at the shake flask level (Erlenmeyer flasks, EMF) and lack a mechanistic understanding of the antibiotic production process, offering poor prospects for successful up-scaling. 

Large-scale fermentations are often performed in multiplication of EMF cultures to obtain enough material for the isolation of the compounds of interest [75]. Since only the transfer into bioreactors allows up-scaling and control of the production process, an initial transfer of cultures into controlled systems, e.g., from EMF into bioreactors, is crucial for successful and economically feasible scale-up. This is especially important as the operating factors like aeration/dissolved oxygen, carbon dioxide, pH, temperature and foam often strongly influence antibiotic production in an unpredictable manner. A proper understanding of these factors is the basis for further successful scale-up strategies [76]. Currently, stirred tank reactors (STR), moving bed and solid-state systems are the most common reactor types applied for fungal biotechnology. For terrestrial fungi, the technical and economic feasibility of large-scale production has been proven for many processes. Whereas STR is predominant in most other industrial fermentation of terrestrial fungi [77], solid-state fermentation is used more widely for production of bioactive compounds [78,79,80,81]. No solid-state fermentation data for marine fungi were found in the literature.

Successful transfer into STR for antibiotic production from marine fungi has been demonstrated e.g., for the production of the tetramic acid compounds ascosetin and lindgomycin produced by an Arctic fungus of the *Lindgomycetaceae* family. The controlled process allowed an increase in product yield (by a factor of 100) and a significant reduction of process time [40]. Other examples for specific approaches to transfer marine fungi into controlled systems were the experiments of Lorenz and Molitoris, who performed cultivation of marine fungi in 20–100-L systems at environmentally occurring high pressures for obligate marine fungi from deep-sea habitats [58]. Application of such specific environmental conditions may stimulate the expression of gene clusters, e.g., for new antibiotic natural products [36].

Only few marine fungal metabolite production processes have been reported in more detail. In the case of *Exophilia pisciphila*, a member of the so-called “black yeasts” isolated from the sponge *Mycale adhaerens*, a 15-L cultivation in a 20-L glass bottle fermenter was carried out with a controlled course of the medium pH, while measuring cell density for the production of the 3,5-dihydroxydecanoic polyester exophilin A [48]. The antibacterial activity of the broth drastically increased after six days and reached its maximum after ten days of cultivation. In another example, the antibiotic activity of the extract of the marine fungus *Hypoxylon oceanicum* from mangrove wood in Shenzen, China, resulted from the production of the well-known macrocyclic polylactones 15G256α, 15G256β, and the novel lipodepsipeptide 15G256γ [38]. In an optimised medium, the cultivation time in 30-L and 300-L reactors was reduced to three days. In this case, production of the metabolites occurred in the stationary phase of growth. Titres of the polylactones and the lipodepsipeptide reached approximately 30 mg/L and 300 mg/L, respectively. Xu et al. studied operating factors such as inoculation, agitation speed, aeration rate, pH control, and nutrient feeding to optimise the production of (+)-terrein, a cyclopentenone from the marine fungus *Aspergillus terreus* (isolated from the sponge *Phakellia fusca*), in a 5-L stirred bioreactor. As for terrestrial bioprocesses, it was recognised that the carbon and nitrogen sources play an important role for compound production [55]. This was also shown for the diketopiperazine chrysogenazine from *Penicillium chrysogenum* originating from a mangrove [44]. For the production of the macrocyclic polyester calcaride A from a *Calcarisporium* sp., a comprehensive study led to a 200-fold increase of compound titres by adaptation of carbon-to-nitrogen ratio, besides the adjustment of the pH regime and changing the mycelial morphology [52]. 

In addition to the typical physiochemical parameters to be controlled, morphology is one of the major parameters influencing the product formation (biosynthesis) and productivity of filamentous organisms. Filamentous fungi have the ability to grow in various morphological appearances, ranging from dispersed filaments to highly dense networks of mycelia, referred to as pellets [82]. As the morphology has impact on the production of the desired product, it becomes highly important for the fermentation process—even though a general optimal form for highest production cannot be stated [83]. Most industrial fermentations are based on dispersed forms, which have to deal with complex non-Newtonian rheological behaviour, finally leading to inhomogeneous conditions [84,85] that affect natural product formation. For instance, effects of cycling oxygen concentrations on penicillin production were shown quite long ago [86]. In contrast, pellet formation would maintain the Newtonian rheological behaviour. However, due to different levels of nutrients and oxygen uptake at several layers of a pellet, pellets reflect a highly inhomogeneous agglomerate of cells on molecular level. As the morphology impacts antibiotic production [87,88], advantages and disadvantages of dispersed mycelia or pellet must be carefully balanced for each biological system [89]. 

Brine cultivation of marine fungi in stainless steel tank reactors may be an additional challenge due to rapid corrosion. Halotolerant marine fungi have evolved unique metabolic mechanisms that are responsive to salt concentrations. These osmoregulatory mechanisms are based on compatible solutes. Since the biosynthesis of these solutes is energetically costly, fungi may exhibit decreased or slower rates of metabolite production in the presence of high salt concentrations. Interestingly, only few reports have investigated the impact of varying salt concentrations on production of antimicrobial marine fungal metabolites, such as the ortho-quinone obioninene from *Leptosphaeria* *oraemaris* [51,54]. These initial findings show that some marine fungal species exhibit increased growth with increasing seawater concentration in the medium while the maximal antimicrobial activity appeared in media containing 25%–50% seawater [54]. Additionally, the osmotic effect on the cell, antibiotic production could be sensitive to the seawater composition, i.e., concentration of specific salts, which could have implications for tank reactor cultivation of marine fungi.

Despite success in some particular cases, many obstacles remain, such as long cultivation times due to slow growth rates, e.g., as of 10 days for maximum production of exophilin A, a 3,5-dihydroxydecanoic polyester [48] or 10 days of cultivation time for the phathalate bis(2-ethylhexyl)phthalate from a *Cladosporium* sp. [51,41]. This holds true for most of the compounds listed in Table 1. Consequently, the production costs would be rather high. 

Inclusion of new methods into process development will significantly contribute to further improvements. Niche-mimic bioreactors might be one opportunity to enhance productivity, but having the industrial scale in mind, adaptation of existing technologies seems to be unavoidable. If adaption is not possible, the development of specialised reactors in industrial scale can be an alternative: The use of immobilised cells in a repeated batch tower reactor led to a significant increase of yield in the production of cephalosporin [90]. Development and fermentation costs must be economically balanced against the value of the product.

Furthermore, many of the above shown studies focused on the chemical isolation of a specific bioactive product without providing bioprocess data or adapting the existing bioprocess conditions, although the scales ranged from 0.3-L to 300-L working volume. Sarkar et al. provided bioprocess data for fermentations of marine fungi for enzyme production. Based on their analysis, there is a demand for further research and development to increase production scales and adapt equipment and processes to the needs of marine fungi (e.g., pressure for deep-sea organisms). They conclude the necessity of further research especially in process development of full fermentative processes [91]. In this context, the increasing knowledge gained by all levels of ‘omics’ techniques (see Section 3.1) provides powerful tools for the biotechnological sector (especially proteomics and fluxomics) to understand and accordingly adapt the respective production properties. Proteomics provide a distinct picture of the presence and activity of all metabolic pathways at specific time points and conditions. This can be used to reveal regulation sites, which can subsequently be addressed by process control and/or genetic modification of the producer. Fluxomics display the conversation of substrate to product, enabling to correlate input, output and losses of a bioprocess [92]. If such fluxes are known, the producing strains could be engineered to change the flux into direction of antibiotic production (see Section 3.2.3).

#### 3.2.2. Production Using Semi-Synthesis

When full fermentative processes cannot be realised in an economically feasible way and the structural complexity limits their chemical synthesis, semi-synthetic processes offer an alternative path in product development. Especially for molecules with complex stereochemistry, the stereosensitivity of enzymatic conversions may be the only way of gaining the optically pure molecule. In semi-synthesis, precursor molecules obtained by a fermentation process are subsequently processed by chemical synthesis or, vice versa, a synthetic product is modified by means of bioconversion using enzymes, whole cells, or even fermentation processes [93]. A prime example for marine fungal natural products as drugs is the discovery of the β-lactam antibiotic cephalosporin C, which was further developed by semi-synthetic approaches: its producer, an *Acremonium chrysogenum* strain, was isolated from seawater samples near a sewer outlet in Sardinia [94]. Although cephalosporin C originally exhibited only weak antibacterial properties, the activity has been gradually increased through the generation of semi-synthetic cephalosporin derivatives [95]. Two-thirds of commercial cephalosporins are derived from biotechnologically-produced cephalosporin C, which serves as a precursor for synthesis of the respective semi-synthetic cephalosporins. After decades of optimisation in fermentation and strain improvement including mutagenesis and genetic engineering (see Section 3.2.3), high-yielding strains of *A. chrysogenum* reach harvest titres of at least 30 g/L for cephalosporin C in fed-batch fermentations [43]. Consequently, despite cephalosporin C being first isolated as early as 1953 [25,26,96], cephalosporins are still indispensable in antibacterial therapy today, including treatment of methicillin-resistant *S*. *aureus*. More than 50 cephalosporins are marketed. In 2009, cephalosporins had a market volume of $11.9 billion, followed by penicillins accounting for $7.9 billion. Together with other β-lactam antibiotics they represent 56% of the world antibiotic market [90]. Despite the rather old and successful history of cephalosporin, no other semi-synthetic approaches have been found in the literature for antibiotics with marine fungal origin. 

The main “competitor” of biotechnological approaches based on full or partial fermentation (semi-synthesis) for production processes is total chemical synthesis. Synthetic organic chemistry is able to produce sufficient amounts for a broad biological screening and to provide access to synthetic analogues for structure-activity relationships (SAR) studies. Major efforts are related to the building of molecules that in nature are produced by metabolic transformations occurring with high yield and rate, and also with high regio-, diastereo-, and enantio-specificity (for state of the art in antibiotic syntheses see [97]). 

One successful example for marine, fungal antibiotics is the chemical synthesis of corollosporine, an antibacterial phthalide derivative from the fungus *Corollospora maritima* isolated from driftwood collected near the Island Helgoland, Germany [46]. Despite the feasibility of organic synthesis, Mancini et al. reported only three natural antibiotic compounds from marine fungi for which total synthesis was described by 2007 [98] and the number has not increased since then. In addition to corollosporin, only farnesylated epoxy cyclohexenones yanuthones A–C (originating from *A. niger* obtained from ascidian *Aplidium* sp.) [99] and pestalone (*Pestalotia* sp. isolated from the brown alga *Rosenvingea*, chlorinated benzophenone) were subjected to total synthesis. In the latter case, biosynthesis studies were considered to support the retrosynthetic approach [34].

The structural complexity of many natural products is the major limiting factor for their chemical synthesis. Obviously, the number of steps needed to build up a compound and the consequent economic feasibility affects their large-scale production drastically. Thus, chemical synthesis represents a non-biotechnological solution for only some antibiotic compounds.

#### 3.2.3. Production Using Heterologous Systems and Genetic and Metabolic Engineering

For the majority of the microorganisms including the untapped resource of unculturable marine fungi, techniques such as metagenomics or genome mining can be used to identify their hidden chemodiversity. Transfecting DNA from the environment to a host strain may allow new natural products to be made [100]. Molecular techniques offer an alternative approach for the (heterologous) expression of “silent” gene clusters and a targeted manipulation of biosynthetic pathways for optimisation of production processes. The use of a heterologous host, which is optimised for industrial-scale production, presents an important tool for bioreactor production instead of a time-consuming optimisation process of cultivation conditions for the native producer strain. Despite the amazing developments in molecular techniques, their application in biotechnological production of natural products is still very limited, due to complexity and size of the genetic clusters encoding for natural products. 

Furthermore, the regulation of antibiotic production is complex and involves multiple regulatory cascades and networks. Knowledge on the regulatory genes can be applied in genetic approaches to activate antibiotic production, but genetic tools for heterologous expression and for homologous manipulation of marine fungi are still in its infancy. In the field of (marine) enzyme production, the strategies have already been applied widely and continue to grow, mainly using the host *Saccharomyces cerevisiae* for increasing yields and changing product specificity, including heterologous protein production [36]. With respect to production of natural products—e.g., antibiotics—there is still a significant need for basic research turning fungal hosts into models for biotechnological purposes [101]. Reports on implementation of metabolic engineering for production of a novel sesterterpenoid (structure not further defined) from the marine fungi *Fusarium heterosporum* and *Aspergillus versicolor* pave the way to that direction [56]. Achievements from the terrestrial field were reported on Tet-on systems, tetracycline-inducible expression systems for quantitative control of gene expression, applied in *Aspergillus niger* [101,102,103]. Some examples of heterologous expression of terrestrial polyketide synthases in model fungal host organisms, namely *Saccharomyces cerevisiae, A. oryzae* and *A. nidulans*, illustrate the feasibility of such approaches [104], which need to be transferred to the marine antibiotic field. Only recently, a successful overexpression of the enniatins in *A. niger*, cyclodepsipeptides originally isolated from *Halosarpheia* sp., demonstrated reprogramming of biosynthetic pathways as a further tool for such approaches [47]. 

Concerning metabolic engineering, the cephalosporin example has paved the way: after decades of optimisation in fermentation and strain improvement high-yielding strains of *A. chrysogenum* has reached the harvest titres of at least 30 g/L for cephalosporin C in fed-batch fermentations [43]. Strain improvement included mutagenesis and genetic engineering. Therefore, homologous cloning of many genes involved in the biosynthetic pathway of cephalosporin synthesis enabled to increase the flux of primary metabolites into secondary metabolite production. It is a common belief among researchers that combinatorial genetic, metabolic, and synthetic engineering as used for the production of cephalosporin C will be the future solution for commercial production of natural compounds.

Metabolic engineering appears to have the potential to be used for large-scale production of marine fungal antibiotics using rationale biochemical designs, but still needs to fulfil its promises in the future. In addition, molecular methods, such as homologous recombination, can be applied to generate mutant strains with altered compound spectra, as demonstrated for the cephalosporin producer [53]. This can overcome the laborious and random driven approaches of UV/chemical mutation. Some examples for marine fungi are available, such as the generation of mutants of a marine *Microascus brevicaulis* using homolog recombination of transcription factors [105] in comparison to a UV approach on the same strain [63]. However, these techniques have to be transferred to the antibiotic field. 

All development of methods using genetic and metabolic engineering can be significantly improved by basing them on knowledge gained by ‘omics’ techniques. Especially RNAseq, revealing the transcription state of a cell in combination with a fully sequenced genome sequence will deliver the starting points for molecular engineering methods [56].

### 3.3. Role of Biotechnology in Downstream Processing 

Downstream processing (DSP) comprises several steps including separation, cell disruption, capture, and concentration steps, as well as extraction, purification, polishing, and formulation. DSP represents the second main part in a production process besides the upstream processing (USP). Unfortunately, only few examples have been reported that focus on the DSP part in marine fungal biotechnology, such as enzymatic treatment, as part of the purification in the cephalosporin production [43].

Despite the fact that DSP is the most expensive and, unfortunately, the most ineffective part of a bioprocess, it is widely underestimated in biotechnological process development [106]. To be able to use, especially in terms of commercialisation, the variety of antibacterial compounds from marine fungi, great efforts must be made in DSP. This includes development of continuous DSP approaches, since biotechnological production is still based on ‘batchwise’ approaches limiting cost- and resource-efficient production. Many other (bio)industries already apply continuous USP and DSP, leading to a reduction in cost, energy, and space requirement [107,108].

Small-scale approaches include a high degree of empiricism, i.e., trial-and-error approaches [109]. However, such a trial-and-error approach is hardly feasible and extremely costly for applications on large-scale. Additional difficulties in up-scaling (e.g., lack of robustness) further limit the transfer of small-scale results to clinical development and any other large‑scale manufacturing. Statistical approaches (e.g., Design of Experiment (DoE)) would speed up such transfers and help to optimise the whole production process [110]. Only efficient and well-understood procedures will lead to a production of sufficient material. There are no standard applications in DSP that are suitable for all types of chemical classes. However, a broad spectrum of downstream tools is available from the established biotechnological processes on compounds from the terrestrial field, which can be easily applied to marine biotechnology. In the future, more integrated marine bioprocesses have to be developed and published.

### 3.4. Role of Biotechnology in Lead Development

Biotechnological methods can also be applied in the late stages of the discovery pipeline, such as lead development. In general, lead development involves medicinal chemistry for the generation of derivatives with improved properties (e.g., better bioavailability and metabolisation) and to enable SAR studies. During lead development, mode of action studies help to understand and improve therapeutic application. Although not biotechnology in a strict sense, Nature already shows how small derivatisations affect bioactivity. One such example is represented by the calcarides, naturally-occurring macrocyclic and linear polyesters from a marine *Calcarisporium* sp. All macrocyclic calcarides inhibit *S. epidermidis* and *X. campestris*, while the very closely related, linear polyesters do not show any activities below a MIC of 100 μM [42]. The number of derivatives produced by marine fungi may occur due to promiscuity of enzymes and/or ecological relevance. Accordingly, biological derivatives can be obtained by bioconversions through enzyme treatment in biotechnological processes. This was successfully demonstrated for the generation of corollosporin derivatives by laccase-catalyzed amination [45]. Likewise, the original fermentative product cephalosporin C in cephalosporin production is enzymatically converted to 7-amino cephalosporanic acid, the key intermediate for the synthesis of the more active semi-synthetic products [111]. In addition to this central derivatisation in the production process of cephalosporins, several other enzymatic conversions have also been used. For instance, certain semi-synthetic cephalosporins, such as cefuroxime, require deacetylcephalosporin as an intermediate [112]. Enzyme-catalysed de-esterification is the only method for the production of this deacetyl intermediate on an industrial scale [113,114]. 

## 4. Conclusions: “Success Factors” for Bridging the Gap from Discovery to Production

Industrial-scale biotechnological processes should be feasible from scientific, technical, and economic perspectives. This viability must be demonstrated through the development of a process concept based on fermentation inclusive of metabolic engineering, purification, molecular design, and synthesis data. Considering the existing knowledge and recent approaches, the following factors seem to be essential for the establishment of more efficient and diverse biotechnological processes for antibiotic compounds of interest:

1. Use of marine fungi. The biodiversity of filamentous fungi from marine sources is mostly untapped. Projects should extend knowledge on both biodiversity and chemodiversity of these unique organisms.
2. Innovation in technology. There is a clear requirement for the “Next Generation Biotechnology”, which includes the new methodological approaches and the understanding of the underlying biological and technological processes.
3. Transdisciplinarity. The transdisciplinary and integrative approach of developmental projects should encompass research and development partners comprising all stages of the early drug discovery pipeline, integrating academia, SMEs, and industry. Thus, important gaps in the knowledge of marine fungi relevant for the production of bioactive compounds should be actively addressed and made relevant to pharmaceutical drug discovery pipelines.
4. Bridging the innovation gap. Researchers can promote innovation directly by employing novel techniques that promote the biosynthesis/production of new metabolites in early stage drug discovery (lead finding) programs and by enriching the pool of metabolites available for screening programs.

The step of bridging the innovation gap seems to have started happening recently. After years of industry-wide disinvestment into antibiotic research, a few large companies have started re-investing in this field. Interesting industry-academia partnerships are being established, such as a Sanofi-Fraunhofer-Gesellschaft partnership to identify and optimise novel naturally-occurring chemical and biological anti-infective compounds. In parallel, political initiatives were started to support and fund the search for new antibiotics, e.g., ADAPT (Antibiotic Development to Advance Patient Treatment), a bill of the US House of Representatives to create an accelerated approval pathway specifically for antibiotics [115].

In summary, marine fungal biotechnology can offer many tools and approaches for successful production of (new) antibiotics and thereby provide an entry point for the sustainable use of marine resources to tackle societal challenges. However, process engineers still face a number of obstacles. They need to be addressed through design of methods for the sustainable development of marine resources as well as by invention of new generation tools and processes to enable a greater understanding of the ocean and its resources. Overall, there are only very limited number of biotechnological processes described for antibiotics from marine fungi. More intensified studies and thorough research efforts are needed to strengthen this promising field. Piggybacking of successful terrestrial processes or other production fields, such as enzymes, can speed up their development. Successful addressing these challenges will require the combined effort of multidisciplinary teams.

## Figures and Tables

**Figure 2:** Overview on all antibacterial compounds from marine fungi that were subjected to biotechnological process developments (covering the literature until March 2016). For groups of compounds only one structure is shown as representative here. Stereochemistry is given, if known from original literature.

**Table:** 

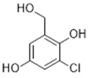	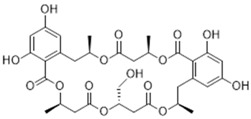	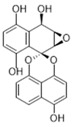
**3-Chloro-2,5-dihydroxy benzyl alcohol [37]**	**15G265α [38]**	**Ascochytatin [39]**
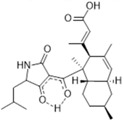	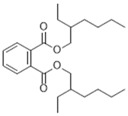	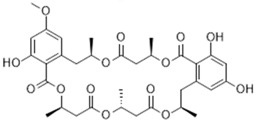
**Ascosetin [40]**	**Bis(2-ethylhexyl)phthalate [41]**	**Calcaride A [42]**
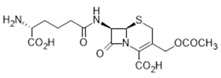	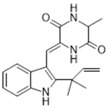	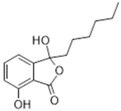
**Cephalosporin C [43]**	**Chrysogenazine [44]**	**Corollosporine [45,46]**
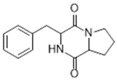	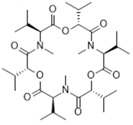	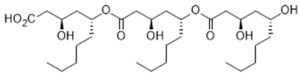
**Cyclo-(Pro-Phe) [37]**	**Enniatin B [47]**	**Exophilin A [48]**
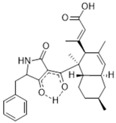	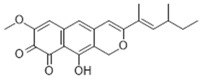	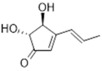
**Lindgomycin x [40]**	**Obioninene [49]**	**(+)-Terrein [50]**

**Table 1:** Realisation of biotechnological approaches for natural product antibiotics from marine fungi, listing all available literature until March 2016. Parameters and fermentation scale were indicated, if available. The origin was stated as concrete as obtained from literature. Abbreviations: Ref., References; EMF, Erlenmeyer flask; STR, stirred tank reactor; MR, Methicillin-Resistant; DSP, Downstream Processing.

Compound, Chemical Class	Producer, Origin	Biotechnological Approach	Antibiotic Activity Against	Ref.
15G265α,β,γ macrocyclic polylactones and lipodepsipeptide	*Hypoxylon oceanicum* LL‑15G256, mangrove	Optimised medium to increase titres Effect of seawater (negative at low temperature) Transfer to Fernbach flasks and 300-L fermenter	*Staphylococcus epidermidis*, *Xanthomonas campestris Propionibacterium acnes*	[38,42]
Ascochytatin, spirodioxynaphthalene	*Ascochyta* sp. NGB4, floating scrap of festering rope collected at a fishing port	Optimisation of medium at small scale	Bacterial two-component regulatory system	[39]
Ascosetin, tetramic acid	Lindgomycetaceae, *Halichondria panicea*, (sponge from Baltic Sea)	Transfer from EMF to STR (10 L): adaptation of medium, increase of yield (factor 100) and decrease of cultivation time	*S. epidermidis*, *S. aureus*, MR *S. aureus*, *P. acnes, X. campestris, Septoria tritici*	[40]
Bis(2-ethylhexyl)phthalate, phthalate *	*Cladosporium* sp., sea water in mangrove area	Transfer from EMF to STR Record of conditions Scaling (2-L fermenter)	*Loktanella hongkongensis*, *M. luteus*, *Rhodovulum* sp., *Ruegeria* sp., *Pseudoalteromonas piscida*, *Vibrio harveyi*	[41,51]
Calcarides A–E, macrocyclic and linear polyesters	*Calcarisporium* sp., Wadden sea water	Biosynthesis study for strain characterisation Biological derivatisation For calcaride A: Adaptation of medium in flasks (13‑fold improvement) STR: 200-fold improvement by pH adaptation, C/N ratio, nature of mycelial growth	Macrocyclic compounds: *S. epidermidis*, *X. campestris* linear polyesters: no antibiotic activity	[42,52]
Cephalosporin, β-lactam	*Aspergillus chrysogenum,* sewage water	Full fermentative optimised process, titres up to 25 g/L Semi-synthesis from 7‑aminocephalosporanic acid (enzymatic) Genetic engineering to reduce by‑products Enzymatic treatment in DSP Immobilised cells in a repeated batch tower reactor	Broad spectrum	[43]
	*Cephalosporium chrysogenum*, sea water	DNA modified by mutagenesis	Broad spectrum	[53]
3-Chloro-2,5-dihydroxy benzyl alcohol, benzene derivative	*Ampelomyces* sp., marine biofilm	Scaling in EMF	*Micrococcus* sp., *Vibrio* sp., *Pseudoalteromonas* sp., *S. aureus*, *S. haemolyticus*	[37]
Chrysogenazine, diketopipera­zine	*Penicillium chrysogenum,* *Porteresia coarctata* (mangrove plant, leaves)	Scaling from 1-L to 5-L flasks Yield of the compound enhanced by modifying the carbon and nitrogen source	*Vibrio cholera*	[44]
Corollosporin and derivates, phthalide derivatives	*Corollospora maritima,* marine driftwood	Biological derivatives by enzymatic treatment Salt dependency of fermentation	*Candida maltosa*, *Escherichia coli*, *Pseudomonas aeruginosa*, *Bacillus subtilis*, *S. aureus, S. aureus* North German epidemic strain, *S. epidermidis*, *S. haemolyticus*	[45,54]
Cyclo-(Pro-Phe), diketopiperazine	Unidentified marine fungus UST030110-009, marine biofilm	Scaling in EMF	Antibacterial antibiofilm: *Micrococcus* sp., *Vibrio* sp., *Pseudoalteromonas*, *S. aureus*, *S. haemolyticus*	[37]
Enniatins, cyclodepsipeptides	*Halosarpheia* sp., mangrove	Heterologous reprogramming of biosynthetic pathways	*E. coli*, *Enterococcus faecium*, *Salmonella enterica*, *Shigella dysenteriae*, *Listeria monocytogenes*, *Yersinia enterocolitica*, *Clostridium perfringens*, *P. aeruginosa*, *S. aureus*	[47]
Exophilin A, 3,5-dihydroxy-decanoic polyester	*Exophiala pisciphila*, *Mycale adhaerens* (sponge)	Transfer from EMF to STR (glass bottle fermenter, 20 L)	*E. facium*, *E. faecalis*, *S. aureus*, MR *S. aureus*	[48]
Lindgomycin, tetramic acid	Lindgomycetaceae, *Halichondria panicea* (sponge from Baltic Sea)	Adaptation of medium Transfer from EMF to STR (10 L): increase of yield (factor 100) and decrease of cultivation time (from 14 to 7 days)	MR *S. aureus*, *S. epidermidis*, *P. acnes*, *X. campestris*, *S. tritici*	[40]
Obioninene, ortho-quinone	*Leptosphaeria oraemaris,* marine driftwood	Effect of salinity on antibiotic production (in EMF)	*Fucus*-associated not identified bacterium	[49]
(+)-Terrein, cyclopentenone	*Aspergillus terreus* PF-26, *Phakellia fusca* (sponge)	Optimisation of operating factors (5-L STR) such as inoculation, agitation speed, aeration rate, pH control and nutrient feeding	*B. subtilis*	[55]
Not determined, sesterterpenoid	*Fusarium heterosporum* and *Aspergillus versicolor*, driftwood and alga	Metabolic engineering	Broad spectrum	[56]
Not determined	*Arthrinium* c.f. *saccharicola*, seawater from a mangrove habitat	Co-culture Stimulation with bacterial elucidators Systematic manipulation of culture conditions: salinity, temperature, pH, and culture medium composition	*Pseudoalteromonas spongiae*, *Vibrio vulnificus*	[57]
Not determined	Obligate fungi, marine deep-sea habitats	High pressure cultivation Scaling 20–100 L	Broad spectrum	[58]

* Although bis(2-ethylhexyl)phthalate is a common plasticizer, its total amount was about 20% of the total fungal extract while hardly any plasticware was used during isolation. It was, therefore, assumed that bis(2-ethylhexyl)phthalate was truly produced by the fungus [41].

## Abbreviations

The following abbreviations are used in this manuscript:

ADAPT	Antibiotic Development to Advance Patient Treatment
DoE	Design of Experiment
DSP	Downstream Processing
EMF	Erlenmeyer Flask
MIC	Minimal Inhibitory Concentration
MIC	Minimal Inhibitory Concentration
MR	Methicillin-Resistant
OSMAC	One-Strain-Many-Compounds
Ref.	References
SAR	Structure-Activity Relationship
SME	Small and Medium Size Enterprises
STR	Stirred Tank Reactor
USP	Upstream Processing
